# Recent Progress of Ophthalmology in France

**Published:** 1875-04-15

**Authors:** Fred. J. Huse


### Translations.

### RECENT PROGRESS OF OPHTHALMOLOGY IN FRANCE.

By Fred. J. Huse, M.D.

I. The Pupil as a Guide During Administration of Chloroform. II. The Ophthalmoscope as a Means of Diagnosis in Acute Phthisis. III. Cystic Degeneration of Conjunctival Mucous Glands.

### I.

M. BUDIN made, in behalf of M. Coyne and himself, a second communication to the Society of Biology in Paris, at the session of Feb. 6th of the present year, upon the pupillary phenomena in asphyxia, and upon the difference between these phenomena in the anæsthesia from chloroform and in the anæsthesia from asphyxia.

He proceeded to state that ever since the first appearance of etherization, the subject of asphyxia has been agitated; respecting this, it was sufficient to refer to the works of MM. Sedillot, Faure, Vulpian, P. Bert and Claude Bernard. That there are manifest in the pupil differences between anæsthesia from chloroform and anæsthesia from asphyxia, he and his colleague were prepared to demonstrate. In the former condition, when it was total, it may be observed that there is contraction with immobility of the pupil. In asphyxia, on the contrary, there is at first a condition of the pupil intermediate between dilatation and contraction, then a very wide dilatation at the time of the appearance of the convulsive phenomena.

When the anæsthesia is attained, excitations fail to produce any modification of the state of the pupil. These phenomena may readily be observed by introducing into the trachea of a dog a canula furnished with a stop-cock, so that the administration of the anæsthetic may be exactly regulated according to the wish of the experimenter.

M. Schiff, however, as well as MM. Bert and Moreau, called in question the result of the experiments already briefly published in this journal.* In giving chloroform to dogs, he had very readily caused their death without having produced pupillary contraction. With chloral, M. Schiff had obtained, it is true, consequent upon an intravenous injection, a contraction of the pupil with total anæsthesia; but when, by means of a sack, he gave chloroform to the animal thus chloralized, he observed re-appearance of the dilatation of the pupil. Chloral, according to his opinion, differed in its action from chloroform; chloroform, moreover, failed in his observations to produce contraction and immobility of the pupil.

MM. Budin and Coyne demonstrated in the following manner that

*Medical Examiner, vol. XV., page 542, and *Chicago Journal of Nervous and Mental Disease*, vol. II., No. 1.

M. Schiff, by administering chloroform by means of the sack, produced in reality not anæsthesia, but asphyxia:

They first injected into the veins of some dogs nearly half a drachm of chloral, sufficient to maintain total anæsthesia during fifteen minutes or more. When each animal was completely anæsthetized they caused him to breathe chloroform vapor freely in the open air; nevertheless, the pupil remained throughout the duration of the experiment (an hour), very firmly contracted and immobile.

Having then performed tracheotomy, they inserted into the trachea of a dog a canula provided with a stop-cock, and when, 45 grains of chloral having been injected, total anæsthesia, accompanied with a contraction of the pupil that became punctiform, appeared, they closed the stop-cock. Asphyxia supervened, and with it a progressive dilatation of the pupils, which at length were very large. Upon opening the stop-cock, the pupils returned to their previous condition; again closing it, they dilated as before.

Thus, *without chloroform*, and with *pure asphyxia*, they reproduced the observations of M. Schiff. Very probably it is owing to some similar defect in the operatory process that contradictory results have been obtained by certain oculists, and by Youngken and M. Maurice Perrin.

As M. Claude Bernard has clearly shown, when the administration of chloroform is mismanaged at the outset, there is a mixture of true anæsthesia and asphyxic anæsthesia. Otherwise, neither chloroform nor ether act as asphyxiants, their vapors exerting upon the nerve centres a direct medicinal influence. All these and many others of the observations of that eminent physiologist of the College of France are confirmed by the experiments of MM. Budin and Coyne.

These authors, however, are far from claiming that asphyxia is the most frequent cause of death in the administration of chloroform and ether; these causes are multiplex, and among them, as MM. Perrin and Lallemand have demonstrated, the most noteworthy, on account of frequence, is syncope.

Asphyxia usually appears in connection with anæsthesia under certain special conditions in the method of administration of these anæsthetics. With other anæsthetics, however, this will not hold true, since it is asserted by MM. Jolyet, Blanche and Claude Bernard (for denial see reports of the session of the Surgical Society of Paris held 3d March, 1875) that nitrous oxide acts invariably as an asphyxiant. It appears that such also is the case with the nitrite of amyl, as M. Bourneville has observed the same pupillary phenomena which appear in simple asphyxia.

In brief, anæsthesia attended with asphyxia causes pupillary phenomena distinctly different from those of simple anæsthesia from chloroform. It is highly important (Sedillot, Claude Bernard) to avoid asphyxia during the administration of chloroform.

Fnally, if in the careful maintenance of anæsthesia with chloroform there appears during the stage of complete insensibility a state of average dilatation, or indeed of any dilatation of the pupil, in place of the usual contraction, the surgeon should at once assure himself whether any cause of asphyxia is influencing the anæsthesia.—*Progres Medical*, No. 7, Feb. 13, 1875.

II.

M. Bouchut recently had, in a case of acute phthisis, simulating typhoid fever, a striking verification of the usefulness of the ophthalmoscope as a means of medical diagnosis, in the advocacy of which he has been one of the earliest and most prominent of the French school, and concerning which his recent communications merit careful attention.*

The case, which is reported in detail† by an interne of the hospital, was that of a girl aged 13 years, who, two weeks before her entry into the hospital, was suddenly seized with a violent pain in the left side, the sense of oppression produced being so intense that two days before her entry she had several attacks of suffocation. There was some headache; a lack of appetite; no diarrhœa or vomiting.

At the time of her admission the most prominent appearance was that of general typhoid condition, with dyspnœa. Obliged to maintain an upright posture, she was breathing painfully at the rate of 70 respirations per minute. The features bore a most manifest expression of extreme debility, there being notable emaciation of the entire body. General hyperæsthesia existed, which was slightly diminished over the thorax—a symptom which was persistent, and upon which special stress is placed in the review of the case—the child crying out at the least pressure upon any portion of its body. The frontal headache caused few complaints. The mind was clear, responses to questions being correct and without hesitation. There was no trouble of the urinary organs. The cardiac sounds appeared normal.

The left anterior wall of the chest yielded upon percussion a slightly more obscure resonance than that of the opposite side, while its elasticity seemed also diminished. In front and on the right side the elasticity and sonorousness were quite normal. Auscultation on the left side revealed, just under the clavicle, a slight, fine sub-crepitant rale; during forcible inspirations and after attacks of coughing, there were some puffs of distant sub-crepitant rales. To the right and in front the vesicular murmur possessed its normal degree. Behind, on the left the respiration was distinct and without any pathological indication; on the right the unfolding of the alveoli was less distinct, the expiration appeared somewhat *soufflante*, and sometimes sub-crepitant rales could be distinguished. The resonance was normal over the entire back of the chest; voice unchanged; temperature 103°; pulse 140. The pupils were dilated and their susceptibility to light was impaired. There was no complaint of any impairment of vision.

Such were some of the more prominent symptoms of a case which was at first diagnosed by M. Bouchut himself as a double pneumonia of a bastard, unusual form. The following day, diarrhœa having set in, there was fresh hesitation and an opinion of its being typhoid fever of a thoracic variety; but the local symptoms were so widely at variance with the general condition, that on the third day M. Bouchut proceeded to make an ophthalmoscopic examination. The pres-

* Medical Examiner, Jan. 15, 1875. *Medical Times and Gazette*, Jan. 23, 1875. *N. Y. Medical Record*, March 27, 1875.

† *Progres Medical*, Feb. 27, 1875.

ence of several tubercules upon both choroids was readily observed by means of the ophthalmoscope, thus affording the indisputable diagnosis of miliary tuberculosis.

Subsequently the thoracic symptoms increased, the rales became more and more marked in intensity as well as extent, and fifteen days after admission the patient succumbed.

At the autopsy, the lungs were found to be *crammed* with the small miliary granules; scarcely a dozen, however, could be found on the peritoneum and intestines, though the mucous membrane, especially of the small intestine, was congested. There were about twenty granulations on the pia mater, and in the left sylvian fissure several were united in a small purulent collection of the size of a pea.

The eyes were divided perpendicularly to their antero-posterior diameter, and disclosed a normal condition of the refracting *media*. Upon carefully raising the retina, the choroidal pigment was found intact, and on each side of the optic nerves there were some whitish, opaque and slightly protuberant points of the size of the head of a small pin. In the right eye there were three of these very near the nerve, and three others at a slight distance. In the left eye there were two in the vicinity of the nerve, and eight others disseminated over the choroid.

While it may perhaps be too much to say, with Cohnheim,* that in miliary tuberculosis the choroid invariably contains tuberculous deposits, still, on the other hand, there is reason to believe, according to M. Bouchut, that such a condition exists more frequently than is generally thought to be the case, and in such a disease as acute phthisis its discovery is of the greatest importance to all parties concerned.

III.

An interesting case of cystic degeneration of the mucous glands of the conjunctiva has recently been described by Dr. Carlo de Vicentiis, in which the conjunctiva presented yellowish irregular nodules upon its tarsal portion of the size of the head of a very small pin, only slightly prominent beyond the general level of the mucous membrane. These nodules were more abundant upon the upper lid than upon the lower, and in the interspaces the conjunctiva was thickened, and also smooth and shining.

A vertical section made through this conjunctiva, after hardening it in alcohol, disclosed, under examination by the microscope, numerous cavities, which were even recognizable with ease by the naked eye, and of which some of the smaller were aggregated together in groups, while others of larger size were isolated. Only a small proportion of them were furnished with an excretory conduit. Their walls were irregular, constituted of fibrous tissue, and they possessed cavities containing the degenerated products of secretion. These contents were composed of granular material and a substance which occurred in yellow scales. The granules were most abundant in the large cavities, while in the smaller ones the scales preponderated.

Dr. Vicentiis considers these cavities as conjunctival mucous glands or glandular cells that have undergone a considerable dilatation and present three stages of degeneration: the

* *Berliner Klin. Wochenschr.* No. 6, 1867.

first, that found in the smallest, still filled with epithelium intact; the second in those of medium size, containing altered epithelium in the form of scales, and finally the granular detritus found in the largest ones.*

* See, however, *Diseases of the Eye*, by Soelberg Wells, 1870, pages 48 to 51.

This lesion is attributed by the author to the retention of the products of secretion, either on account of too much secretory activity or by reason of obstruction of the excretory conduits.—*Movimento Medico-Chirugico di Napoli*, and *La France Medicale*, 13th March, 1875.

